# Principles and Practice of Medicine

**Published:** 1871-12

**Authors:** 


Essentials of the Principles and Practice of Medicine. A handbook for Students and Practitioners, by Henry Hartshorne, A. M., M. D. Third edition thoroughly revised. Philadelphia: H. C. Lea, publisher.

This little volume is already well and favorably known through its former editions, and is generally acknowledged to be the most comprehensive and useful hand-book ever offered to the profession.

---

The Endemic and Epidemic Diseases of Mobile, their Causes and Prevention, by Jerome Cochran, M. D.

---

Annual Report of the Surgeon-General of the United States Army.

---

Three Cases of Imperforate Anus, with remarks by J. H. Pooley, M. D., Yonkers, N. Y.

